# Glycobiology of Reproductive Processes in Marine Animals: The State of the Art

**DOI:** 10.3390/md10122861

**Published:** 2012-12-17

**Authors:** Alessandra Gallo, Maria Costantini

**Affiliations:** Laboratory of Animal Physiology and Evolution, Stazione Zoologica Anton Dohrn, Villa Comunale, Naples 80121, Italy; E-Mail: alessandra.gallo@szn.it

**Keywords:** embryo development, ferilization, glycobiology, oogeneesis, spermatogenesis

## Abstract

Glycobiology is the study of complex carbohydrates in biological systems and represents a developing field of science that has made huge advances in the last half century. In fact, it combines all branches of biomedical research, revealing the vast and diverse forms of carbohydrate structures that exist in nature. Advances in structure determination have enabled scientists to study the function of complex carbohydrates in more depth and to determine the role that they play in a wide range of biological processes. Glycobiology research in marine systems has primarily focused on reproduction, in particular for what concern the chemical communication between the gametes. The current status of marine glycobiology is primarily descriptive, devoted to characterizing marine glycoconjugates with potential biomedical and biotechnological applications. In this review, we describe the current status of the glycobiology in the reproductive processes from gametogenesis to fertilization and embryo development of marine animals.

## 1. Introduction

Glycoconjugate chemistry and glycobiology were rapidly evolving as scientific disciplines in the 1980s. Their impact, however, on understanding fundamental biological processes was not immediately forthcoming. In recent years, there has been resurgence in the field with major discoveries leading to powerful new insights into the complex role of glycoconjugates in biological processes. Progress in glycobiology has shed light on a range of complex biological processes associated with, for example, disease and immunology, molecular and cellular communication and developmental biology [1]. The diversity of complex carbohydrates has fascinated and frustrated glycobiologists for years. The most studied for their biological properties are mammalian sulfated polysaccharides or glycoconjugates constituted by glycosaminoglycans (GAGs) composed of negatively charged acidic chains, most of them covalently linked to proteins [2]. Glycoconjugates are also widely diffused in marine environment. In fact, they are responsible for a remarkably diverse array of biological activities in marine organisms ranging from immunity, sperm-egg binding, thrust generation in nonflagellar cyanobacteria, electroreception in sharks, and a multitude of cell-cell and cell-molecular recognition events [1]. For these reasons, there is a considerable interest in characterizing the glycan structures that mediate many of these processes, as many will have potential medical and biotechnological applications. Moreover, manipulating oligosaccharide composition in the embryo promises new insights into their developmental functions [3]. For example, *O*-linked *N*-acetylglucosamine (*O*-GlcNAc) is a highly dynamic post-translational modification of cytoplasmic and nuclear proteins. Although the function of this abundant modification is yet to be definitively elucidated, considering also that all *O*-GlcNAc proteins are phosphoproteins. Further, the serine and threonine residues substituted with *O*-GlcNAc are often sites of, or close to sites of, protein phosphorylation. This implies that there may be a dynamic interplay between these two post-translational modifications to regulate protein function. It has been demonstrated that several cellular function and developmental regulation might be affected by changes in *O*-GlcNAc levels [4]. In this respect, one of the most fascinating properties of complex carbohydrates moieties is their enormous information potential, considering glycocoding as an information management system in reproduction and embryonic development. In fact, reproduction is an evolutionary imperative and a fundamental feature of all known life. Understanding of the mechanism(s), by which the spatial/temporal regulation of early development is managed, represents a very topical issue. The “transitions” which occur during cell to cell cluster, cell cluster to early organ architecture, and early organ architecture to functional organ development are fundamental and mirror the evolutionary process of biological information management. All of these “transitions” involve increasing complexity, the development of hierarchies of information management systems (integrated bidirectionally), and spatial/temporal regulation, which relies on historical events to map future structure and function.

Carbohydrates appear to be important functional groups in reproductive biology with variation in glycosylation products contributing toward reproductive functionality in male and female animals [5,6]. Carbohydrates are known to play essential role in various biological processes including development.

In this review, we will present the relevance of glycobiology to gametes physiology, fertilization and embryo development. 

## 2. The Glycobiology in Oogenesis

During oogenesis, immature oocytes are arrested at the prophase I of the meiotic process and are characterized by a large nucleus that is referred as the “germinal vesicle”. Along the maturation process oocytes grow and acquire the competence for fertilization. During the growth, large amounts of glycoconjugates, in particular glycoproteins, are synthesized and represent the molecular constituents of cortical vesicles, vitelline envelope and yolk granules of the fully grown oocyte of marine organisms [7,8].

Cortical alveoli are membrane-limited round structures located in the oocyte cortex containing neutral glycoproteins, carboxylated glycoconjugates, neutral glycoproteins plus sialic acid-rich glycoprotein, and neutral glycoproteins plus sulfated glycoconjugates [9].

The major constituent of cortical alveoli of fish eggs is the hyosophorin (HSP), a highly glycosylated protein in which carbohydrates account for 80% to 90%. Polysialoglycoprotein (PSGP) is the first HSP isolated from the unfertilized eggs of rainbow trout (*Salmo gairderi*) [10]. Homologs were subsequently found in salmon [11,12] and flounder [13] and they are collectively denoted as hyosophorins, which respond to the following criteria: high carbohydrate content and tandem repeats of an identical peptide sequence. 

PSGP from rainbow trout eggs has a low protein content (about 15% by weight) and a high sialic acid content (about 60% by weight) most of which occurs in polysialic acid (polySia) chains linked to *O*-glycosidic carbohydrate units [14]. PSGP consists of tandem repeats of tridecaglycopeptide carrying three *O*-linked glycans with polySia chain which is an α2,8-linked polySia with chain length of up to 25 sialic acid residues [15]. These polySia chains are capped at the non-reducing ends by deaminated neuraminic acid, 3-deoxy-D-glycero-D-galacto-nonulosonic acid (KDN) [16] and their biosynthesis is developmentally regulated and occurs at later stage of oogenesis [17]. A species-specific structural diversity has been revealed in polySia chain of salmonid egg PSGP.

In rainbow trout and in *Oncorhynchus* fish species, PSGP sialic acid residues are exclusively *N*-glycolylneuraminic acid (Neu5Gc) while *Salmo* and *Saluelinus* fish species contain both *N*-acetylneuraminic acid (Neu5Ac) and Neu5Gc residues [14,15,18]. Hyosophorin isolated from flounder has a neutral fucosylated pentaantennary glycan chain [13] that in *herring* and *Fundulus heteroclitus* is sialylated.

In fish, cortical alveoli contain a considerable cellular heterogeneity of glycoconjugate sugar residues. The glycoconjugate pattern is specie specific, being cortical alveoli rich in α-*N*-acetyl-D-glucosamine (α-GlcNAc), sialic acids, α-*N*-Acetyl-D-galactosamine (α-GalNAc), α- or β-GalNAc, β-D-Galactosyl-(1-3)-*N*-acetyl-D-galactosamine (β-D-Gal(1-3)-GalNAc) in swordfish [8]; GalNAc and α-galactose (αGal) in flatfish [19]; GlcNAc, GalNAc, Gal and sialic acid in protogynous teleost *Epinephelus marginatus* [20] and GlcNAc, GalNAc and sialic acid in bluefin tuna [7]. 

The cortical alveoli of fishes, therefore, are considered homologous to the cortical granules present in invertebrates [21,22] and in other vertebrate species [23,24,25]. Several studies on the contents of cortical granules in sea urchins oocytes demonstrated that they contain diverse repertoire of molecules that includes enzymes, such as an ovoperoxidase, a glycoprotein containing both mannose (Man) and GlcNAc moieties [26], a protease, and a glycosidase; structural proteins such as SFE9 (soft fertilization envelopes clone 9), proteoliaisin, and SFE1 (soft fertilization envelope clone one); glycosaminoglycans; and perivitelline molecules such as glucanase and 330 kDa fibrillar glycoprotein hyaline [27].

**Figure 1:**
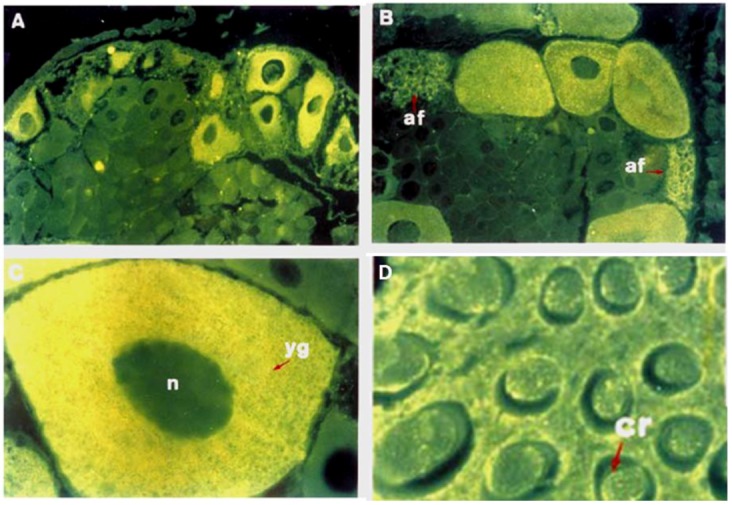
Immunolocalization of shrimp ovarian peritrophin (SOP) in the shrimp ovarian sections at different stages of oocyte development. Immunofluorescence labeled previtellogenic (**A**) and vitellogenic oocytes (**B**) localized at the periphery of ovarian lobe. Higher magnification revealed that SOP was specifically present in the oocyte cytoplasm from vitellogenic (**C**) and late vitellogenic (**D**) ovarian stages; no label was detected in the nucleus or in the surrounding follicular cells. In vitellogenic oocytes, SOP immunoreactivity was also detected in CRs (**D**) (Modified from [35]).

In Crustaceans, mature oocytes are characterized by the presence of rod-like bodies, called cortical rods (CRs), arranged radially around the periphery of the oocyte plasma membranes [28,29,30,31,32] and located in the extracellular crypts formed by the invagination of the oolemma into the egg cortex. During spawning, CRs are released upon contact of the eggs with seawater and form a jelly investment around the eggs in many penaeiod shrimp species but is lacking in other crustaceans [30,31,33]. Biochemical characterization of the isolated CRs has revealed that they contain approximately 25%–30% carbohydrates and 70%–75% proteins by weight [29]. Histochemical evidence has also consistently demonstrated that CRs are glycoprotein-based materials [34]. To date, two CRs glycoproteins have been characterized: the shrimp ovarian peritrophin (SOP) (Figure 1) [35,36] and the cortical rod protein (CRP) [37,38]. SOP and CRP are high-Man glycoproteins that also contain a minor proportion of GlcNAc, sialic acid and fucose (Fuc) residues, some of which are additionally sulfated, playing a key role in modulating shrimp sperm acrosomal reaction response [39].

One of the most important aspects of oogenesis is the formation and storage of the yolk proteins, a process known as vitellogenesis. In oviparous animals, yolk proteins are the most important source of nutrients for developing embryos, constituting 60%–90% of the total egg proteins. Oocyte yolk proteins derive from the enzymatic cleavage of a common precursor called vitellogenin (Vg), which is a glycolipophosphoprotein showing similar characteristics in vertebrates and invertebrates [40]. 

In all model systems studied, Vg is uptake by the growing oocyte, in which is proteolytically cleaved into a characteristic suite of yolk proteins: a high-density lipoglycoprotein called lipovitellins, highly phosphorylated phosvitins, and a β-component, stored within membrane-bound structures called yolk platelets, major source of nutrients for eggs and developing embryo [8]. The primary degradation products of Vg have also been shown to play a role in regulating oocyte hydration [41] and buoyancy of eggs [42]. In addition, the ion-binding properties of Vg serve as a major supply of minerals to the oocytes [43].

The structure of the glycan moieties of Vg has been discovered only in decapod crustaceans. Recent study of the composition of oligosaccharides attached to the Vg of the crayfish *Cherax quadricarinatus* showed that Vg, which is produced in the hepatopancreas and secreted to the hemolymph [44], is posttranslationally modified by *N*-linked oligosaccharides. These *N*-glycan moieties are composed of monoglucosylated and highly mannosylated glycans, ranging from Glc1Man9GlcNAc2 (one glucoses, nine mannoses, and two GlcNAc) to Man_5_GlcNAc_2 _(five mannoses and two GlcNAc). Vg is a large hydrophobic protein containing glucose-capped oligosaccharides. It has been suggested that glycosylation of Vg plays an important role in the folding and subunits assembly to achieve the mature protein in the hemolymph and ovary [45]. Glycosylation is known to increase solubility of proteins [46] so *N*-glycans might have a significant role in keeping this large, hydrophobic protein in the hemolymph to improve its transport in the ovary [45]. The uptake of Vg into the oocytes is known to be mediated by receptors [47,48]. In this respect, the glycan moiety might play a role in recognition and receptor-mediated endocytosis. Once taken into the oocytes, it might also have a role in packaging and compacting its products in yolk bodies [45].

In several sea urchin species, Vg is synthetized in the intestine [49] as a 195-kDa glycoprotein and secreted into celomic fluid of the adult. From the coelom, this glycoprotein is absorbed by the nutritive phagocytes (accessory cells) of the ovary, where it is stored. Then it is transported to the growing oocytes to be accumulated as a 180-kDa glycoprotein, termed major yolk protein (MYP) [50,51,52]. This decrease in molecular weight from Vg to MYPs seems to be a common phenomenon in sea urchin. It is possible that Vg is slightly modified in molecular structure after its incorporation into the gonads. Shyu *et al.* [49] presumed that this modification takes place in the oocytes, but it has also been suggested that it occurs in the nutritive phagocytes of both sexes immediately after incorporation [51].

Glycosphingolipid (GSL) is composed of a sugar chain and a ceramide consisted of a fatty acid and sphingoid base [53]. GSLs are ubiquitous on the outer surface of the plasma membrane in animal cells and play an essential role in intercellular interaction and recognition [54,55,56,57]. However, GSL functions have not yet been clarified because of the structural complexity of the sugar chain and ceramide moiety [58,59]. According to the structure of the carbohydrate moiety, GSLs have been divided in two main groups: neutral glycosphingolipids (NGSLs) and acidic glycosphingolipids.

Gangliosides are more complex glycosphingolipids in which oligosaccharide chains, containing sialic acid, are attached to a ceramide. In the eggs of some sea urchins, the chemical structures of the major gangliosides have been identified as NeuGcα2-6Glcβ1-1Cer (M5) and HSO3-NeuGcα2-6Glcβ1-1Cer (T1). M5 ganglioside constitutes more than 90% of the egg gangliosides and 0.8% or more of the egg dry weight [60,61,62]. In unfertilized egg ganglioside M5 has been shown to be localized in the plasma membrane and in yolk granule, where it is associated with yolk lipoproteins and is involved in the uptake of yolk lipoproteins into the growing oocytes during oogenesis and transported from yolk granules to other cellular components during embryogenesis. M5 ganglioside associating with yolk lipoproteins in yolk granules may be considered a significant stored material to be utilized for early embryogenesis [63,64,65].

Sea urchin oocytes contain a variety of NGLs such as glucosylceramide, melibiosylceramide, ceramide trihexoside, and GL-5 [61,66,67]. The chemical structures of these glycolipids was determined showing that ceramide moieties of these are almost identical. 

Glucosylceramide (Glcβl-1Cer) and melibiosylceramide (Galα 1-6Glcβ1-1Cer) have the same long-chain base compositions that are very characteristic, all of them are phytosphingosines. The fatty acid compositions of these glycolsphingolipids also resemble each other [68].

The chemical structure of the ceramide trihexoside was determined (Galβl-6Galβ1-6Glcβ1-lCer) and its carbohydrate structure is a novel trisaccharide. Glucosylceramide resembles ceramide trihexoside in ceramide structure suggesting that it is synthesized from glucosylceramide [66].

GL-5 chemical structure is Fucα1-3GalNAcβ1-4(Fucαl-3)GlcNAcβl-4Glcβ1-lCer. GL-5 has a unique saccharide sequence: the reducing terminal disaccharide core is GlcNAcβl-4Glc. The defucosylated core structure (GalNAcβ1-4GlcNAcβl-4Glcβ1-) is a novel trisaccharide chain. Fuc directly binds to GalNAc and the sugar structure is one of the shortest saccharide chains so far found among the difucosylated glycolipids [67,69]. Kubo *et al.* [68] speculate on the possible biological functions of these glycolsphingolipids suggesting that they could participate to sperm egg interaction process.

Another feature of oocyte development is the formation of an extracellular eggshell, or vitelline envelope [70], that in some species is encompassed by a jelly-like layer. The most common molecules on the surface of oocyte envelope are glycoproteins [71,72,73,74] that are provided by the liver [75] or by the ovary [76,77,78] or by both of them [79]. The vitelline envelope possesses many functions, such as prevention of polyspermy, protection of the growing oocyte and the developing embryo, uptake of nutrients and other molecules during oogenesis, and guidance of the spermatozoa to the oocyte. 

The vitelline envelope of fish eggs is composed of an outer layer rich in glycolipids and of an inner layer, called zona radiate, containing neutral glycoproteins [7]. The zona radiata is composed of 3–4 glycoproteins derived from glycoproteins precursors, known as choriogenins [8]. During oogenesis, choriogenin is synthesized in either the ovary or the liver and incorporated into the egg envelope [80]. 

Although the biological function of these glycoconjugates is unknown, it has been suggested that they are involved in the hormones binding, in the transport of metabolites and ions across plasmalemma, and in the sperm-egg interaction process [81]. 

From the vitelline envelope of the unfertilized eggs of rainbow trout (*Salmo gairdneri*), it was isolated, for the first time, a new acid glycoprotein that is designated as “KDN -glycoprotein” because it contains only KDN but no sialic acid as the acid carbohydrate moieties. Other carbohydrate components of KDN-glycoprotein are Gal and GalNAc [18]. KDN-glycoprotein consists of 500 kDa polypeptide chain, to which a number of *O*-linked glycans are attached. These oligosaccharide chains have a core trisaccharide Galβ1-3GalNAcα1-3GalNAc in which the terminal Gal residue is blocked by a single residue of KDN and the proximal GalNAc residue is linked to α2,8-linked oligo(KDN) chains with different degrees of polymerization [82]. KDN-glycoprotein was shown to contain also *N*-linked complex-type glycan chains as minor components. KDN-glycoprotein is the unique glycoprotein of the rainbow trout egg envelope but it is also the major component of vitelline envelope of chum and kokanee salmon. KDN-glycoprotein is located in second layer of vitelline envelope but exposed to the outer surface of vitelline envelope around the micropyle through which spermatozoa can penetrate the oocyte at fertilization. Although, little is known about the functional roles of these glycan chains, probably they act as cell surface receptor for spermatozoa in fertilization [83]. An analogous family of mucin-type glycoprotein that contains Neu5Gc instead of KDN is the major component of cherry salmon vitelline envelope [84].

Glycoconjugates forming zona radiate of different teleost fish eggs have been well investigated. In the flatfish *Solea senegalensis*, the presence of glycans with terminal GalNAc and/or αGal and with terminal/internal αMan is reported. As oogenesis proceeds, the glycan pattern of zona radiata decreases drastically and in the last phase of maturation only few βGalNAc residues are detected. Zona radiate of dusky grouper *Epinephelus marginatus* is characterized by a higher content of GlcNAc and sialic acid [20] as in swordfish zona radiate in which α-L-Fuc residues are also detected [8]. During oocyte development, the glycan pattern changes and this may reflect the different activity of zona radiate during different phases of oogenesis [19].

Studies carried out on different species of Crustaceans, such as rock shrimp *Rhynchocinetes types* and peneaid shrimp *Sycionia ingentis*, have characterized oligosaccharides on the glycoproteins present on oocyte envelope [85,86]. They show a high concentration of glucose (Glc) and α1-3mannose (α1-3Man) on the oocyte at all levels of maturation. Gal, Fuc, GlcNAc and GalNAc are others monosaccharides detect in lowest concentration on the oocyte envelope. Fuc, Gal and GalNAc exclusively have a structural function

In glycoconjugates while α-GlcNAc has great importance, together with Glc and Man, during gamete interaction process, in spite of its small concentration.

Differently from Crustaceans, α-GlcNAc is highly expressed on the oocyte envelope of ascidian *Phallusia mammillata.* This monosaccharide, as well as GalNAc, is predominant with 86% by weight of total sugar content and Fuc, Man and Glc accounted for the remaining 14% [87]. GalNAc, α-GlcNAc and Man, with Gal are also the major components of the vitelline coat of another ascidian *Halocinthia roretzi*, in which monosaccharide analysis of glycans reveals also a high content of arabinose, xylose and rhamnose, but any information about their plausible biological significance is not available. Glycans are *O*-linked and Gal and xylose residues are detected at reducing termini. Several analysis suggest that 1,4-linked xylose, 1,3-linked Gal and GalNAc residues constitutes the core structures of anionic glycans [88]. Rhamnose is also one of the monosaccharides detected in the vitelline coat of another ascidian *Ciona intestinalis* [89]. The vitelline coat of the mature oocyte of *Ciona intestinalis* contains three glycoproteins and the most abundant carbohydrate is GalNAc, followed by Fuc and Gal [90]. 

In different sea urchin species, the oocyte jelly coat was shown to contain two major acid glycoproteins, sialic acid-rich glycoprotein and fucose sulfate glycoconjugate (FSG) [91,92]. The sialic acid-rich glycoproteins have a low protein content and contain many *O*-linked sialyglycan chains attached to threonine residues on the core protein. The polySia chains in polysialic acid-glycoprotein contain Neu5Gc residues and are composed of Fuc, Gal, and GalNAc residues. Most interestingly, the inner residue linkages in the polySia chains contain unique (→5-*O*_glycolyl_-Neu5Gcα2→)*_n_*, linkages [93]. This structure represents the first naturally occurring polySia as an integral part of a glycoprotein, which potentiates the FSG-induced acrosome reaction in a dose dependent manner [94]. FSGs are the major macromolecules present on the egg jelly coat responsible for inducing the sperm acrosome reaction [95,96,97]. They are linear polysaccharides with a repeated unit composed of sulfated 1–4 hexose moieties. It was found that, among sea urchin species, they differ in sugar composition (L-Fuc or L-Gal), glycosidic linkage (α1→3 or α1→4) and in the sulfation pattern (2- or 4-*O*-sulfate) [98,99]. These differences are responsible of species-specific induction of the acrosomal reaction [94,98].

Starfish egg jelly coat contains two glycoproteins: the first one is a high Man glycoprotein with a molecular mass of 80 kDa and an unique saccharide structures, but its function is not well clarified [100,101]; the second one is a highly sulfated Fuc-rich glycoprotein, a unique glycoprotein playing a key role in the trigger of sperm acrosome reaction, named acrosome reaction-inducing substance (ARIS) [101,102].

The biological activity of ARIS resides in one of the sugar fragments called fragment 1 [94,103]. The molecular size of this glycan is about 10 kDa and does not contain amino acid residues. Its structure was revealed as ten or so repeats of the following pentasacharide unit; [→4)-β-D-Xyl*p*-(1→3)-α-D-Gal*p*-(1→3)-α-LFuc*p*-4(SO_3_^−^)-(1→3)-α-L-Fuc*p*4(SO_3_^−^)-(1→4)-α-L-Fuc*p*-(1→] [104]. This sugar chain links to the peptide part by *O*-glycosidic linkage trough another sugar chain with different structure from fragment 1 [94].

The inner sugar portion of ARIS has been isolated as fragment 2. It is mostly composed of sulfated glycans and retains about 10% (w/w) of the protein part. Fragment 2 has a molecular mass of 400 kDa and its glycans are *O*-linked. It is composed of the heptasaccharide units of [→3)-Gal*p*-(1→3)-Fuc*p*-(1→3)-Gal*p*-(1→4)-GalNAc*p*-(1→4)-GlcNAc*p*-6(SO_3_^−^)-(1→6)-Gal*p*4(SO_3_^−^)-(1→4)-GalNAc*p*-(1→] [105]. Polymerizations of outer glycans are necessary to form the layer. ARIS has another acid sugar chain composed of Man, GalNAc and GlcNAc, although it is not important for the biological activity [106]. 

The oocytes of a tropical abalone *Haliotis asinina* have two protective barriers: the egg jelly coat and the vitelline envelope. The egg jelly contains two major glycoproteins of 107 kDa and 178 kDa, whereas the vitelline envelope contains a broad spectrum of protein bands ranging from 15 to 200 kDa. Glc is the major sugar residue of both egg jelly and vitelline envelope glycoproteins, whereas minor proportions of arabinose, fructose, Gal, and Fuc are present in both the egg jelly and vitelline envelope [107]. 

## 3. The Glycobiology in Spermatogenesis

Spermatogenesis is a developmental process during which spermatogonial stem cells produce highly differentiated spermatozoa. It starts with the mitotic proliferation of spermatogonia and formation of primary spermatocytes, passing then through meiosis into secondary spermatocytes, which differentiate into spermatids and finally through spermiogenesis into mature spermatozoa [108,109,110,111].

During spermatogenesis, glycoprotein composition significantly changes suggesting the important role of these components in this process [112,113,114]. In sperm development, some complex and not well known germ cell-somatic cell interactions are involved and several reports suggest that glycoconjugates are involved in cell adhesion during this process [115,116].

Spermatogenesis in fishes is characterized by a cystic mode that consist of a set of Sertoli cells surrounding a group of synchronously differentiating germ line cells through the respective developmental stages until spermatogenesis is completed and mature spermatozoa are formed. The development of spermatogenic cells requires a special microenvironment that is created by the Sertoli cells [117]. Through cytoplasmic projections, these cells surround clones of primordial germ cells forming cysts in the seminiferous tubules [118].

The testes of the teleosts have two compartments: a tubular compartment constituted of Sertoli’s cells and germinative cells forming spermatogenic cysts; and an interstitial compartment that is formed of connective tissue and Leydig’s cells [119].

The identification and localization of glycoprotein oligosaccharide sequences in the testis of teleost fish *Solea senegalesis* have been well investigated [120]. Glycoproteins are localized in both compartments of the testis, the interstitial and germinal compartments, but also in the basal lamina separating them. In the interstitial stroma, a very complex glycoprotein composition, including asialo-, as well as sialoglycans, in *N*- and *O*- linked oligosaccharides and *N*-linked glycans, was discovered. It has been also demonstrated the glycosylation pattern of the interstitial stroma is depending on the testicular region analyzed. In fact Neu5Acα2,3Galβ1,4GlcNAc and GalNAcα1,3(L-Fuc α 1,2)Galβ1,3/4GlcNAcβ1 are more abundant in the medullar region than in the cortex. This different pattern is due to a differential glycoprotein compound trafficking patterns from the vascular system to each testis region. This occurs because germ cell proliferation and differentiation take place in the cortical region of the testis while final sperm maturation in medullar region, in order to support distinct glycoprotein requirements in different phases of developmental process. 

The interstitial compartment of fish species is characterized by the presence of melano-macrophage centers, that contain glycans terminating with Galβ1,3GalNAc but their role is not known, and Leydig cells, that unlike from other teleost species, does not exhibit glycoprotein oligosaccharide sequences [121].

The basal lamina is characterized by glycans with terminal/internal Man, internal βGlcNAc, terminal Neu5Acα2,6Gal/GalNAc, Neu5acGalβ1,3GalNAc, Galβ1,3GalNAc, Galβ1,4GlcNAc, GalNAc, αGal and αL-Fuc. This complex glycosylation pattern is related to the presence of meshwork composed of several glycoconjugate components, responsible for biological function of basal laminas.

In the germinal compartment, Sertoli cells express sialoglycans terminating with Neu5Acα2,3Galβ1,4GlcNAc that may be involved in the establishment of interaction between adjacent spermatocysts playing a key role in the organization of spermatogenic cysts.

Different from *Senegalese sole*, the Sertoli cells of other fish species, such as spotted ray and Nile tilapia, display a more complex glycosylation pattern, but sialoglycans seem to be lacking in those species [121,122]. 

Glycoprotein oligosaccharide sequences are absent in spermatogonia while primary spermatocytes express, in cytoplasm and nucleus, glycans terminating with Galβ1,3GalNAc and αGalNAc, respectively [120]. Since germ cell nuclear glycoproteins are associated with the chromatin, it has been suggested that they play a role in the regulation of transcription factors and in the control of cell cycle [123]. Spermatid cytoplasm exhibits *N*-linked glycans, containing high-Man residues, as well as oligosaccharides terminating with α/βGalNAc, αGal and αL-Fuc. These glycoproteins were also found in fine granular structures located in the cytoplasm of spermatids.

The presence of glycoconjugates has been also demonstrated in germ cells and somatic cells, Leydig and Sertoli cells, in cartilaginous fish testis. In particular, germ and somatic cells undergo extensive modifications regarding the composition of glycoside residues at the level of surface, cytoplasm, and nucleus. On the surface of germ and Sertoli cells, it has been identified cadherins, glycoprotein superfamily containing mannosyl chains. Cadherin play a key role in establishing interactions between germ and somatic cells, implied in cyst and spermatoblast formation. In addition, germ cells and Sertoli cells undergoing apoptosis change the cellular surface composition, specifically overexpressing on the cell surface GalNAc and Gal that make them susceptible to phagocytosis. During cyst maturation, also Leyding cells change their surface glycoconjugate composition due to a modification of their activity during spermatogenesis. Germ cells express new sugar residues during their differentiation, the presence of glycoconjugates is limited to the Golgi zone in spermatocytes, to acrosome formation region in spermatids, and to the acrosome in spermatozoa [121]. This finding suggests that the acrosome glycosylation pattern depends on stoichiometric and spatial distribution of binding sites and/or the activity of glycolytic enzymes and glycosyltransferases [121,124]. Glycoconjugates are also expressed in the germ cell nucleus where they are involved in the control of cell cycle [121].

Studies on sea urchin testis have revealed the presence of the yolk protein precursor, Vg, in immature male gonad as well as in the female one, suggesting that male Vg could be a precursor of MYP incorporated in the testicular nutritive phagocytes as a nutrient source for spermatogenesis [51]. As spermatogenesis proceeds, MYP decreases in quantity; in fact, it is utilized as material for synthesizing new substances that participate to the formation of spermatozoa and are metabolized as an energy source during this period [125].

The immature sea urchin testis also contains a large quantity of polysaccharides, most of which is probably glycogen in the form of granules [126,127]. The polysaccharide content decreases as gametogenesis proceeds in both sexes, since possibly they are used as an energy source, as suggested for *Strongylocentrotus intermedius* [127].

In sea urchin testis, it has also been demonstrated the expression of a heavily sialylated glycoprotein, named flagellasialin. Flagellasialin has a molecular mass ranging from 40 to 80 kDa and contains a unique polySia sulfated α2,9-linked polyNeu5Ac [128]. Flagellasialin is exclusively located to the flagellum where probably α2,9-linked polySia regulates voltage sensitive sodium and calcium channels influencing the intracellular calcium and sperm motility [129,130]. In the flagellum but also in the sperm head, it has been demonstrated the expression of a 190 kDa glycoprotein linked to α2,8-linked polyNeu5Ac structures. The biological function of this glycoprotein is not yet clarified. The conformational differences between α2,9- and α2,8- linked polySia structures and their co-localization in the same sperm might reflect functional differences of these two polySia-containing glycoprotein at fertilization. The α2,8-linked polyNeu5Ac structure occurs also in glycolipids, localized in the sperm head in order to facilitate rearrangement of the membrane proteins on the sperm surface upon sperm activation [131,132]. 

In Decapoda it has been shown that immature germ cells and spermatozoa have different glycoconjugate composition. In *Aristaeomorpha foliacea*, immature germ cells only express *N*-linked oligosaccharides which contain terminal and internal α-D-Man, internal β-D-GlcNAc, NeuAcα2,6Gal/GalNAc and terminal NeuAcα2,3Galβ1,4GlcNAc. Spermatozoa express in the cytoplasm both *N*- and *O*-linked oligosaccharides. The *N*-linked oligosaccharides consist of terminal GlcNAc while *O*-linked oligosaccharides terminated with β-Gal(1-3)-GalNAc and/or α-GalNAc in the cytoplasm, whereas they ends with sialic acid linked to β-Gal(1-3)-GalNAc in the nucleus [133]. The extracellular matrix in which the spermatozoa are embedded consists of neutral and acid glycoconjugate. Acid polysaccharides have been reported in *Albunea symnista* [134] and *Panulirus homarus* [135]. Neutral glycoprotein and acid polysaccharide have been found in *M.rosebergii* [136]. Differences in glycoconjugate composition of extracellular matrix between immature and mature hemispermatophores have been observed: the former expresses both *N*- and *O*-linked glycoconjugates while mature hemispermatophores express only *O*-linked oligosaccharides and contain more sialyl-glycoconjugates than immature ones [133]. The role of extracellular matrix in decapods is not well known. It has been suggested that the acid mucopolysaccharide of spermatophores may act as a cementing agent or an antimicrobial agent [137] or in the maintenance of spermatozoa during the storage within female spermathecae [133,138].

Previuos study on *A. foliacea* also demonstrates that sperm glycoprotein pattern seasonally changes and spermatozoa undergo maturative changes in glycoconjugate compotion during their transit from testis to hemispermatophore [139]. 

## 4. The Glycobiology in Fertilization

Fertilization is a highly specialized process of cell-cell interaction that marks the creation of a new individual. Although the details of fertilization vary between species, it generally consists of five major events. The first event is the recognition and the species-specific interaction between sperm and the egg coat. This interaction allows spermatozoa to undergo the acrosome reaction, an exocytosis of the acrosomal vesicle located on the tip of the spermatozoa head, which releases a lytic agent. These events enable spermatozoa to cross the extracellular matrix and reach the oocyte plasma membrane where binding between the two cells occurs and causes the fusion of the genetic material of spermatozoa and egg leading to the activation of egg metabolism, mitosis and the beginning of development [140,141,142].

Glycoconjugates play a number of pivotal roles in multifaceted processes during fertilization [1,143]. The initial interaction between oocytes and spermatozoa involves glycoproteins of oocyte envelope and complementary sperm surface receptors [144]. In particular, the terminal oligosaccharides of egg envelope glycoprotein are responsible for spermatozoa binding through recognition of species-specific polypeptide chains on the spermatozoa [87,145,146].

The first evidence supporting the function of complex glycoconjugates in fertilization was found in sea urchins [91]. Two carbohydrates are involved in sea urchin fertilization. The first carbohydrate is egg jelly coat FSG that binds a glycoprotein receptor, named suREJ located exclusively in the plasma membrane just on the sperm acrosomal vesicle at the anterior apex of the spermatozoa head [147,148]. suREJ contains one epidermal growth factor module and two *C*-type lectin carbohydrate- recognition modules [147]. After this binding, the spermatozoa undergo consecutive morphological and biochemical changes called the acrosomal reaction. In response to signals transduced by sperm receptor, acrosomal vesicles fuse with the plasma membrane and actin polymerizes to form the acrosomal process. The exocytosis of acrosomal vesicles expels the protein bindin that coats the spermatozoa acrosomal process [149]. The second carbohydrate involved in sea urchin fertilization is a glycoprotein receptor for bindin in the vitelline layer [150]. This receptor, namely EBR1, is a 350 kDa glycoprotein containing both *N*- and *O*-linked oligosaccharide chains with different biological activity, being inactive and active respectively. The active oligosaccharide is linked to serine or threonine via an *O*-glycosidic bond to GalNAc, which contain a Gal bound β(l→3) to the linkage sugar. The remainder of the structure consists of GlcNAc and Fuc residues, with the sulfate moieties on the Fuc residues. The *O*-linked oligosaccharide chains show different level of sulfation correlated to their activity, being greatly enhanced with increasing sulfation. Two-step models involving carbohydrate and protein chains have been proposed for sperm-egg interaction in sea urchin. The first step is postulated to be a low-affinity ionic interaction of the sulfated *O*-linked oligosaccharide chains of the receptor with spermatozoa that is not species specific. This is followed by a high affinity, species-specific interaction of one or more domains of the polypeptide chain and bindin on the acrosomal process [151]. The species specificity of this interaction is determined by differences in the position of attachment of the oligosaccharide chains on the protein backbone [152].

In starfish, the acrosome reaction is induced by the combination of ARIS and other two components of the egg jelly coat: sulfated steroid saponin namely Co-ARIS and an oligopeptide known as asterosap [153,154]. When spermatozoa reach the egg jelly coat, the first binding occurs between ARIS and its receptor, specifically located to the anterior region of spermatozoa heads [155]. 

The tertiary structure of the ARIS is important for its activity. The signal for a correct folding is derived from fragment 1. The fragment 1 has a double helix and a compact carbohydrate core with sulfates protruding in pairs away from the center of the helix. It has been demonstrated that the double helix structure of ARIS saccharide chain is important for the induction of acrosome reaction [94]. The binding gates calcium channels inducing an intracellular calcium increase. This signal causes exocytosis by which spermatozoa expose the devices essential for penetration through the egg coats and for fusion with the egg plasma membrane.

In sea water, ARIS requires Co-ARIS to induce acrosome reaction [156] and microdomain changes [103]. Co-ARIS is composed of sulfated steroid and a pentasaccharide chain. Its activity requires sulfate moiety and mainly depends on the structure of steroidal side chain, while not necessarily requiring a specific structure of the saccharide chain [157]. Spermatozoa do not have a specific receptor for Co-ARIS. It has been suggested that steroid ring and side chain of Co-ARIS may infiltrate or be inserted into spermatozoa plasma membrane and the sulfated group and sugar chain contribute to keep it in the right position and orientation to interact correctly with other components of spermatozoa plasma membrane [101]. 

Asterosap is a glutamine-rich tetratriacontapeptide with a 25-residue ring formed by a disulfide linkage that is essential for its biological activity [158]. It transiently increases the intracellular pH and calcium via the activation of asterosap receptor that is a guanylyl cyclase located in the sperm flagellar plasma membrane [159,160,161]. Calcium and pH increase are essential to trigger acrosome reaction. 

In archaeogastropods, the molecules analogous to sea urchin bindin and EBR1 are lysin and its receptor, most extensively studied in abalone. The abalone spermatozoa swims easily through the egg jelly coat and reaches the vitelline envelope. The contact between the spermatozoa and the vitelline envelope induces the polymerization of actin to generate acrosomal process and the exocytotic of acrosomal vesicle that release protein lysin onto the surface of the vitelline envelope. Lysin is a 16 kDa nonenzymatic, cationic protein that forms a dimer, which is able to bind a giant 1000 kDa glycoprotein named Vitelline Envelope Receptor for Lysin (VERL) [151]. VERL is a long, unbranched, fibrous glycoprotein that is composed of at least 50% saccharide, containing Glc and Man residues, and comprises 30% of the vitelline envelope mass [162]. Dimer binding results in monomerization and the tight, species-specific binding of lysin to VERL that involves the carbohydrate moieties of VERL linked to serine or threonine via an *O*-glycosidic bond. Upon binding lysin monomers, the fibrous VERL molecules lose cohesion and splay apart, through which the spermatozoa passes to reach the egg cell membrane and the tip of its acrosomal process fuses with the egg plasma membrane [163,164]. Other vitelline envelope glycoproteins of 30–50 kDa, even if do not binding lysin, could be involved in mediating species selectivity based on their interaction with VERL [162,165].

In ascidians, sperm-egg binding is mediated by an enzyme-substrate complex established between a specific spermatozoa surface glycosidase and corresponding glycans on the surface of the vitelline coat [166,167]. In the alkaline seawater, this complex remains stable, since the glycosidase has an acid pH optimum [167,168,169]. In *Ascidia nigra* and *Phallusia mamillata*, *N*-acetyl-glucosaminidase in sperm membranes recognizes terminal α-GlcNAc residues on the oocyte envelope [168,170], while in *Ciona intestinalis* and *Halocynthia roretzi*, the sulfated, Fuc-containing glycans of vitelline coat glycoproteins are responsible for the spermatozoa binding to the vitelline coat. In particular, the sperm glycosidase, α-L-fucosidase, binds to terminal L-Fuc residues of the vitelline layer [94,101,160,171]. The degree of sulfation and the proper spacing of sulfate groups seem essential for biological activity [88]. When spermatozoa bind vitelline envelope, acrosome reaction occurs, the acrosomal outer membrane fuses with the plasmalemma enclosing the acrosome, resulting in exocytosis of the acrosomal substances. After the acrosome reaction, apical processes protrude mainly from the peripheral region of the apex of the spermatozoa head [172,173,174]. Although the chemical nature and the precise role of the acrosomal substances remain to be elucidated, it has been proposed that these acrosomal substances are responsible for membrane fusion between the apical processes and the egg plasma membrane [173,174]. The sperm-egg vitelline interaction activates a spermatozoa lysine system. In particular, three proteases are involved in spermatozoa penetration of the vitelline coat: two trypsin-like proteases, acrosin and spermosin, and one chymotrypsin-like protease. The chymotrypsin-like activity is involved in spermatozoa penetration of the vitelline coat, but spermosin and acrosin both function to increase the rate of fertilization [175,176,177].

In decapod crustaceans, the first sperm-egg interaction is established between the apical end of a sperm’s appendage (spike) and the outermost egg envelope. In particular, in *Rhynchocinetes typus* it has been demonstrated the presence of a lectin-like molecule on the tip of spermatozoa spike that recognized specific carbohydrates on the oocyte envelope such as GlcNAc, Glc and Man [86,178]. At this point, the tip of the rigid spike exerts a lytic effect upon vitelline envelope causing a perforation through which the spermatozoa passage to reach the plasma membrane oocyte [179,180,181].

Polyspermy is the fusion of more than one spermatozoa with an egg, which is a lethal condition to the embryos of most organisms [182]. At fertilization, rapid and slow changes occur to block polyspermy. The rapid changes are related to the modification of egg membrane potential. The slow changes are due to the modifications of egg vitelline envelope, which transform it to a hard layer called the “fertilization membrane”. The hardening of vitelline envelope is trigged by the contents of cortical granules that are released upon fertilization. Fertilization membrane forms a protective barrier that repels additional spermatozoa, but also bacteria and small eukaryotic invaders [27]. Several studies report that glycoconjugates are implied in to block polyspermy.

In sea urchins, fertilization envelope formation is initiated by trypsin-like proteases that cleave both a sperm-binding protein [183,184,185,186] removing supernumerary spermatozoa and preventing further spermatozoa binding, and the proteins that connect the vitelline envelope to the plasma membrane [27,187]. At the same time, sugars that are released from the cortical granules attract water into the perivitelline space allowing the vitelline envelope lifts off the egg plasma membrane that is also due to the formation of hyaline layer [27]. Hyalin is a filamentous molecule that tends to form aggregates forming an amorphous layer [188]. In particular, its carbohydrate residues act as receptors for hyaline-hyalin and hyaline-cellinteraction [27]. Then, ovoperoxidases harden the vitelline envelope that becomes resistant to both mechanical and enzymatic modifications [27]. Ovoperoxidase released in the perivitelline space interacts with another cortical granule protein, the proteoliaisin. When this complex is associated with the nascent fertilization envelope, ovoperoxidase catalyzes the covalent cross-linking of juxtaposed tyrosine residues in adjacent polypeptide chains, including SFE 1 and SFE 9, to form a stable, macromolecular complex [189]. Proteoliaisin targets the ovoperoxidase to the nascent vitelline layer/fertilization envelope [190] and protects this enzyme from proteolytic digestion [191]. 

Fish spermatozoa lack an acrosome and they attach directly to the egg plasma membrane through the micropyle, a single, small pore in the chorion [148]. Following sperm-egg fusion, cortical alveoli fuse with the plasma membrane and discharge their contents, PSGP, hyaline, proteases and transglutaminases into the presumptive perivitelline space [15,182,192,193,194]. Once released from the cortical alveoli, proteases cleave the 200 kDa PSGP (L-PSGP) into small 9 kDa PSGP (H-PSGP) [15,192,193]. Both amino acid and carbohydrate compositions of L-PSGP and H-PSGP are identical. H-PSGP is still too large to permeate the chorion. This establishes a colloid osmotic pressure, which causes an influx of mostly external water that swells the perivitelline space. The perivitelline space cushions the embryo and bathes it in a special medium containing the 9 kDa PSGP, lipids, carbohydrates, and ions as well as providing a sink for nitrogenous wastes [195,196,197]. The perivitelline space also provides room for the free movement and growth of the embryo, which, due to its hypertonicity, absorbs water osmotically and slowly swells during embryogenesis [198]. With the establishment of the perivitelline space, the rise in hydrostatic pressure is aided by the hardening of the chorion, which contributes to the closure of the micropyle and thereby reduces the probability of polyspermy and microbial infection [199,200]. The hardening process is catalysed by transglutaminases activated of PSGP that have proteinase activity [201]. Thus, activated transglutaminase forms covalent ε-(γ-glutamyl)-lysine crosslinks constituents of adjacent proteins or glycoproteins of chorion [202]. 

Ascidian eggs lack cortical granules and prevent polyspermy by first releasing a large quantity of glycosidase, followed by an electrical modification of the egg plasma membrane [87,203,204,205]. Sperm-egg binding triggers eggs to release large quantities of glycosidase that rapidly bind the vitelline-coat surface as spermatozoa surface glycosidase blocking the binding of supernumerary spermatozoa [74,101,204].

## 5. The Glycobiology in Embryo Development

Glycoconjugates have relevance for embryo development, tissue and cell specialization and organogenesis. Several studies reported that glycoconjugates are involved in embryo development of marine invertebrates. Early studies on inhibition of glycoprotein synthesis and embryonic development of sea urchin revealed that tunicamycin (a mixture of homologous antibiotics that blocks *N*-glycosylation of proteins, inhibiting *N*-acetylglucosamine transferases) blocked the process of gastrulation [206,207]. Subsequently, it was established that the sensitivity of the gastrulation process to tunicamycin could be related to a marked increase in the level of *N*-linked glycoprotein synthesis, which occurs just before gastrulation [208]. This requirement for glycoprotein synthesis was consistent with the finding that gastrulation could also be blocked by inhibiting synthesis of dolichyl phosphate [209,210], the lipid carrier required for oligosaccharide chain assembly in *N*-linked glycoprotein synthesis. These early studies of the effect of tunicamycin on development also revealed that this drug blocked spiculogenesis when added at the late gastrula stage [206]. More recently, this apparent requirement for *N*-linked glycoprotein synthesis during spicules formation has received greater attention. Carson *et al.* [211] established that addition of a monoclonal antibody (mAb 1223) to a culture of primary mesenchyme cells caused a block in spiculogenesis. Immunofluorescence studies showed that the 1223 antigen was primary mesenchyme cell-specific. Immunoblot analysis revealed that one of the proteins containing the 1223 epitope was a 130-kD polypeptide whose level of expression correlated with the acquisition of the ability of primary mesenchyme cells to accumulate calcium during spicule formation. These observations, coupled with subsequent studies establishing that the 1223 antigen was a glycoprotein, and that the mAb 1223 was directed toward a carbohydrate chain on the glycoprotein [212,213], led to study both the distribution of this epitope in the embryo and the nature of the oligosaccharide moiety in more detail. These studies have established that the epitope on the glycoprotein recognized by mAb 1223 is a complex, *N*-linked oligosaccharide chain [213] that is found in the cortical granules of the egg, disappears after fertilization and reappears in association with primary mesenchymal cells before spiculogenesis [212,214]. Moreover, some authors [215] demonstrated that two other independently generated monoclonal antibodies, 1G8 [216] and B2C2 [215] also recognized an oligosaccharide group on the same 130-kD protein, suggesting that it is a highly immunogenic mesenchymal cell marker.

**Figure 2:**
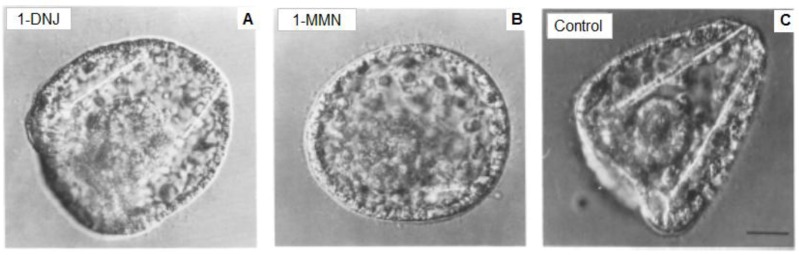
Effects of glycoprotein processing inhibitors. Eggs were fertilized and embryos cultured until 72 h post fertilization, when they were treated with (**A**) 4 mM 1-deoxynojirimycin (1-DNJ, which blocks both glucosidase I and II; (**B**) 2 mM 1-deoxymannojirimycin (1-MMN, which blocks mannosidase I); (**C**) untreated controls. The samples were fixed in 2% glutaraldeyde. Scale bar, 5 μm. (Modified from [217]).

Given the complex nature of the carbohydrate chain of the 1223 antigen and its apparently essential role in a step in spiculogenesis, it seemed likely that inhibitors of the processing of oligosaccharide chains would block spicule formation. Kabakoff and Lennarz [217] reported the results of biochemical and morphological studies with such inhibitors using both intact embryos and primary mesenchyme cells in culture. In order to ascertain whether the processing of high mannose oligosaccharides to complex oligosaccharides is necessary for spiculogenesis, intact embryos and cultures of spicule-forming primary mesenchyme cells were treated with two glycoprotein processing inhibitors, deoxymannojirimycin (1-MMN) and deoxynojirimycin (1-DNJ). In both cases, normal embryonic development between gastrula and prisma stage was affected by impairing or completely blocking spicule formation (Figure 2). 

These inhibitors did not affect gastrulation in whole embryos or filopodial network formation in cell cultures. It was also demonstrated that (a) 1-MMN entered the embryos and blocked glycoprotein processing in the 24-h period before spicule formation as assessed by a twofold increase in endoglycosidase H sensitivity among newly synthesized glycoproteins upon addition of 1-MMN; (b) 1-MMN did not affect general protein synthesis until after its effects on spicule formation were observed; (c) Immunoblot analysis with an antibody directed towards the polypeptide chain of the 130-kD protein (mAb A3) showed that 1-MMN did not affect the level of the polypeptide that is known to be synthesized just before spicule formation; (d) 1-MMN and 1-DNJ almost completely abolished (>95%) the appearance of mAb 1223 reactive complex oligosaccharide moiety associated with the 130-kD glycoprotein. 

These results indicate that the conversion of high mannose oligosaccharides to complex oligosaccharides is required for spiculogenesis in sea urchin embryos and they suggest that the 130-kD protein is one of these essential complex glycoproteins. In fact, a correlation between a block in processing of the oligosaccharide chain of glycoproteins and impairment of spicule formation was established. Moreover, the conversion of high mannose ologosaccharides to complex oligosaccharides was required for spiculogenesis in sea urchin embryos, suggesting that the 130-kD protein was one of these essential complex glycoproteins. 

**Figure 3:**
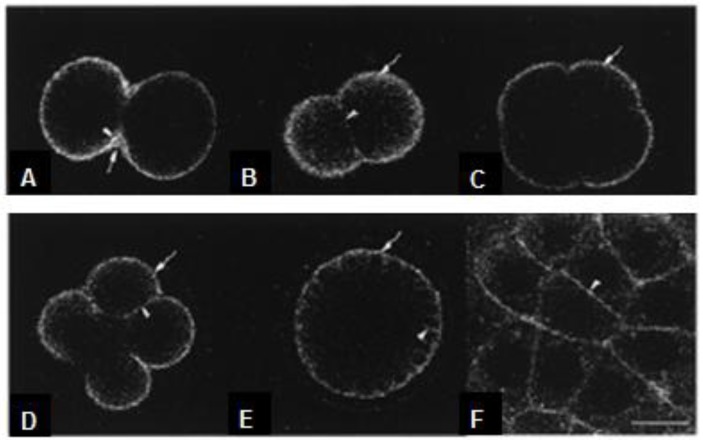
Detection of NBD-M5 in early development with a confocal microscope. To observe the localization of NBD-M5 in living embryos, fertilized eggs pre-labeled with NBD-M5 were grown in artificial sea water without dye. At the end of mitosis (first cell division), the thick ECM (hyaline layer) near the cleavage furrow was stained (**A**); similar tick layers of staining were observed in 2- (**B**), 4- (**C**), 8-cell stage (**D**) and blastula before hatching (**E** and **F**). (**F**) is an higher magnification of the cortical region of (**E**). The localization of NBD-M5 did not change. Scale bar, 10 μm. (Modified from [62]).

Gangliosides play important roles also during embryo development. In fact, revealing the distribution of M5 during embryogenesis is an important step in evaluating the significance of gangliosides in early development. Nezuo *et al.* [62] reported that ganglioside M5 was secreted during embryogenesis and localized in the extracellular matrix (ECM). Since M5 exists in unfertilized eggs in yolk granules, it was transported from the yolk granules to the ECM and/or the plasma membrane after fertilization. M5, however, is quantitatively constant during embryogenesis [218]. Using fluorescent-labeled ganglioside NBD-M5, similar thick layers of staining were observed in 2-, 4-, 8-cell stage and blastula embryos (see Figure 3). A number of studies have been shown the role of gangliosides as mediators in the interaction of various cells with ECM in vertebrates, showing also a high affinity to fibronectin [219,220]. Since the sea urchin embryo is surrounded by several ECM components, such as molecules similar to vertebrate fibronectin, collagen and laminin [221], M5 may be involved in the organization of these molecules, although it remains to be determined whether M5 actually interacts with these proteins. 

Dermatan sulfate is a macromolecule member of a class of natural, structurally complex, sulfated, linear polymers named glycosaminoglycans (GAGs). There are many types of GAGs generally grouped into four categories: (1) hyaluronic acid or hyaluronan; (2) keratan sulfate; (3) chondroitin sulfate (CS)/DS; and (4) heparan sulfate (HS)/heparin. They are biosynthesized as polysaccharides of repeating disaccharides with an *N*-acetylhexosamine, *N*-acetylgalactosamine (GalNAc), or *N*-acetylglucosamine as one of the sugars. The alternating sugar is glucuronic acid (GlcA) with the exception of keratan sulfate, which instead contains galactose. The hyaluronic acid is not further modified, whereas the other classes are modified by (1) the addition of *O*-sulfate groups on various hydroxyls (the three classes); (2) 5-epimerization of some GlcA residues to form iduronic acid (IdoA) residues (DS, HS, heparin), and (3) the removal of acetyl residues from some hexosamines replaced with *N*-sulfates (HS and heparin) [222].

Unfertilized eggs of the sea urchin *Strongylocentrotus purpuratus* are surrounded by a gelatinous layer rich in sulfated fucan. Shortly after fertilization this polysaccharide disappears, but 24 h later, the embryos synthesize high amounts of dermatan sulfate concomitantly with the mesenchyme blastula-early gastrula stage when the larval gut is forming [223]. This glycosaminoglycan has the same back-bone structure [4-α-L-IdoA-1→3-β-D-GalNAc-1]*_n_* as the mammalian counterpart but possesses a different sulfation pattern. It has a high content of 4-O and 6-*O*-disulfated galactosamine units. In addition, chains of this dermatan sulfate are considerable longer than those of vertebrate tissues. Adult sea urchin tissues contain high concentrations of sulfated polysaccharides, but dermatan sulfate is restricted to the adult body wall where it accounts for 20% of the total sulfated polysaccharides, indicating that it retains a biological role in the adult stage. Vilela-Silva *et al.* [223] concentrated their studies on the characterization of the sulfated polysaccharide synthesized by the larvae of the sea urchin *S. purpuratus*. Shortly after fertilization, the sulfated fucan of egg jelly disappears, and by 24 h of development the embryos begin to synthetize large amounts of dermatan sulfate. In addition, sulfation at the 4-*O*-position decreases markedly in the dematan sulfate from adult sea urchin when compared with the glycan from larvae. The importance of dermatan sulfate for the development of sea urchin embryos was emphasized in several experiments where inhibitors of sulfation, chain elongation, or proteoglycan formation were added to the medium and inhibited embryonic development [207,224,225,226,227]. 

Interesting data were reported on the very attractive process during embryo development, the transdifferentiation, also known as lineage reprogramming. In fact transdifferentiation means the conversion of cells from one differentiated cell type to another. Reber-Muller *et al.* [215] demonstrated that transdifferentiation at high rates is also initiated by treatment of the isolated tissue fragments with the monoclonal antibody (mAb)19 developed against the ECM of the medusa *Podycoryne carnea*. This antibody interferes strongly with cell-ECM interactions. Moreover, oxidation of carbohydrates with sodium-meta-periodate in enzyme linked immunosorbent assays (ELISAs) demonstrated that mAb19 is directed against a carbohydrate epitope. The results suggested that in striated muscle of the medusa cell adhesion and the maintenance of the differentiated state depend on carbohydrate mediated cell-ECM interactions. If these interactions were disturbed by mAb19 the striated muscle cells will undergo DNA-replication and transdifferentiate. In conclusion, medusa cells depend on the presence of species-specific carbohydrates for cell adhesion and spreading [216]. 

## 6. Conclusions

Marine environment offers a tremendous biodiversity and original polysaccharides, presenting a great chemical diversity that is largely species-specific. Marine polysaccharides present an enormous variety of structures, presenting a real potential for natural product drug discovery and for the delivery of new marine derived products for therapeutic applications. They are involved in a range of complex biological processes associated with diseases, immunology, molecular and cellular communications, and mainly with developmental biology. In fact, most recent findings showed that glycobiology of reproductive processes in marine organisms represent a rapidly advancing field and permits an innovative view on the carbohydrate-based interactions that lead to gametogenesis, to fertilization and to embryo development. In conclusion, this study is of special interest for general scientists in reproductive biology and medicine, for glycobiologists and for clinicians working in the fields of human and animal fertility. 
